# Enhancing GaN LED Efficiency through Nano-Gratings and Standing Wave Analysis

**DOI:** 10.3390/nano8121045

**Published:** 2018-12-13

**Authors:** Xiaomin Jin, Simeon Trieu, Gregory James Chavoor, Gabriel Michael Halpin

**Affiliations:** Electrical Engineering Department, California Polytechnic State University, San Luis Obispo, CA 93407, USA; simeon.trieu@gmail.com (S.T.); gjchavoor@calpoly.edu (G.J.C.); gabriel.halpin@gmail.com (G.M.H.)

**Keywords:** GaN, LED, nano-grating

## Abstract

Based on our recent work, this paper reviews our theoretical study on gallium nitride (GaN) light-emitting-diode (LED). The focus of the paper is to improve LED light extraction efficiency through various nano-grating designs. The gratings can be designed at different locations, such as at the top, the middle, and the bottom, on the LED. They also can be made of different materials. In this study, we first present a GaN LED error-grating simulation model. Second, nano Indium Tin Oxide (ITO) top gratings are studied and compared with conventional LED (CLED) using standing wave analysis. Third, we present results related to a patterned sapphire substrate (PSS), SiO_2_ Nanorod array (NR), and Ag bottom reflection layer. Finally, we investigate the nano-top ITO grating performance over different wavelengths to validate our design simulation, which focusing on a single wavelength of 460 nm.

## 1. Introduction

Gallium nitride (GaN) based light-emitting-diodes (LEDs) continue to prove themselves increasingly useful in the world of solid-state lighting. Although highly efficient, scientists continue to investigate ways to increase their internal and external quantum efficiencies. In general, the efficiency of an LED is limited by the maximum angle that light can escape from the surface as defined by Snell’s law. Since GaN has a high refractive index compared to air, light can only escape the LED if it approaches the surface within +/− 23° of the normal incidence. The large difference in the refractive index of air and GaN results in a low critical angle. Therefore, the low critical angle traps light within the LED, reduces light output, and increases the device temperature. Improving the light extraction efficiency of GaN LEDs has been approached by several different ways. Early approaches for improvement of light efficiency include surface roughness or texturing [1]. Then, adding a material with a refractive index between the indices of GaN and air to the LED surface increases the limiting angle [2]. Photonic crystal top grating [3] and nano pillar Multiple-quantum-well (MQW) [4] have also been proposed. Recently, several research groups have used various gratings as methods for increasing light output and placed these gratings either on the surface [5], the bottom [5,6], or the sidewalls [7] of the LED.

Fabrication techniques have improved in the recent years. They have allowed design structures, such as periodic top gratings [8,9,10,11,12,13], patterned sapphire substrates (PSS) [14,15,16,17,18], and reflection layers (R) [19,20,21,22,23], to improve the external quantum efficiency by enhancing light extraction. One study showed that moving an Ag-based reflective mirror from below to above the sapphire substrate increases light extraction by 21% [24]. Recently, a SiO_2_ nano-rod array (NR) was also used in GaN LED [25,26,27,28]. As technology progresses, more complex structures are designed using a multi-nano structure in a single GaN LED. For example, in 2018, GaN LEDs with a hybrid structure were fabricated and studied, which were a combination of sidewalls and microhole arrays [26]. In general, a cost-effective method to design high efficiency GaN LEDs is still highly desired. A more comprehensive GaN LED design simulation or theoretical study, including several nano-stuctures, is very valuable in this case before the fabrication.

Currently, no study fully examines or directly compares the above several light extraction efficiency (LEE) improvement methods theoretically. For example, which method is more efficient, and whether they can be combined. How does an Ag reflector affect light extraction in those structures? In this paper, we present comprehensive simulation results of Indium Tin Oxide (ITO) top nano-grating, and a direct comparison of GaN LED with a patterned sapphire substrate, SiO_2_ nano-rod array, and Ag bottom reflection layer. Additionally, we find that among all of the above technologies, NR has more potential of improving the light extraction efficiency.

## 2. GaN LED Grating Simulation with the Error Grating Model

### 2.1. Basic Structure of GaN LED

The GaN LED models used in this paper were built through 2D finite difference time domain (FDTD) analysis. We used FDTD to provide a solution to Maxwell’s equations by using Yee’s mesh, a system in which the E- and H-field components are solved based on the previous spatial E- and H-field components. FDTD also solves Maxwell’s equations on a point by point basis and can accurately simulate the effects of nano-gratings in the LED, such as reflection due to linear dispersion or total internal reflection, transmission of escaping light from the LED, and scattering at the grating. Since FDTD decomposes space and time into separate components, the model is meshed into small cells whose side length must be much smaller than the wavelength of light to obtain accurate results. Using this method, we can simulate the average power emission of LEDs.

**Figure 1:**
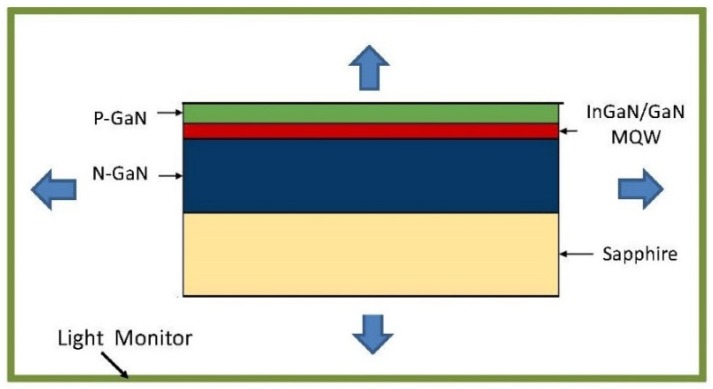
Structure and basic simulation model of conventional Gallium nitride (GaN) based light-emitting-diode (LED).

As shown in Figure 1, light is generated in the multiple quantum well (MQW) region between the positive and negatively doped GaN regions of the diode. Since the computer model simulates light propagation in the LED, we assign all light to emerge as a continuous wave (CW) from the middle of the MQW region. The model is built as a 2D representative section of the whole LED and implements a uniform light distribution across the MQW region. The four green bars around the edge of the LED in Figure 1 are the light monitors. They measure the intensity of light emerging from top, bottom, and sides of the LED. The top monitor always sits 560 nm above the top non-grating layer of the LED, 100 nm higher than the tallest grating triangles. The bottom and side monitors sit 100 nm from the LED body. These positions were held constant throughout this research to make sure that each set of simulations has only limit variables that can change the intensity of the light emitted from the LED.

The conventional GaN LED model, as shown in Figure 1, consists of a p-GaN layer, InGaN/GaN MQW, n-GaN layer, and sapphire substrate, whose thicknesses and refractive indices are listed in Table 1. The sapphire substrate thickness was set to 80 μm.

### 2.2. Error Grating Model

**Figure 2:**
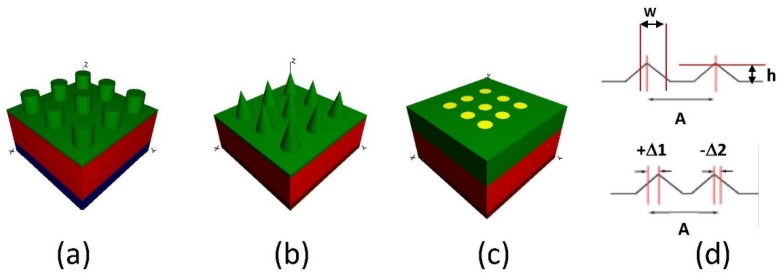
The schematic diagrams of the top grating simulation, (**a**) cylindrical pillar grating, (**b**) conical pillar grating, and (**c**) cylindrical nano-hole grating. (**d**) Error grating model: Normal reference grating model and error grating model with both positive and negative shifts.

Usually, it is not very practical to fabricate all kinds of textures or patterns to select the optimized structure [29,30]. Therefore, we first simulated three typical gratings: Cylindrical pillar grating, conical pillar grating, and cylindrical nano-hole grating. Figure 2a–c are illustrations of the top grating implementing above three grating types. Additionally, we found that the conical pillar grating is more efficient compared to the cylindrical pillar grating, and a small grating period will yield a better light extraction efficiency [31,32]. In the grating simulation model, for top-grating, bottom grating, and nano-hole grating, there are three major parameters that affect the light extraction: The grating period (*A*), grating height (*h*), and bottom width (*w*).

We also proposed a top and bottom grating model with each cell randomly shifted a distance along the axis in varying degrees of randomization intensity to further understand the effects of fabrication defects on the top and bottom gratings, as shown in Figure 2d [5]. Usually, the widths of holes can be fabricated to great precision. Often the placement of holes causes concern, as it shifts the grating location and affects light extraction efficiency of otherwise ordered photonic crystal structures.

The error grating model still makes use of the 2D FDTD method. Random displacements in position form the basis of the error grating model with a normal grating as a reference. Displacements can move either direction from the grating cell’s original center point. The error grating model shows examples of a positive and negative Δ*x* shift. This randomization then applies to all grating cells in the photonic crystal arrangement with Equation (1):

*x*_pos_ = *N* × period + (2 × rand −1) × *R* × period (1)

where *N* is an integer index defining the original grating cell location, the period is the grating period (A), rand is a pseudo-randomly generated number from 0 to 1, and *R* is the randomization factor from 0 to 1. The quantity, Δ*x*, in Figure 2d represents the (2 × rand − 1) × *R* × period in Equation (1). By varying *R*, which applies to all grating cells, from 0 to 1 in 40 steps, the individual rand factor can be emphasized or deemphasized. This process repeats for each of the grating models to calculate the light extraction efficiency variation.

The randomization creates local variation of the grating structure, *A* and *w*. Our simulation of several top and bottom grating shows that randomization in gratings appear to help the light extraction efficiency, peaking at about a randomization factor of R = 10% in most simulations [5]. In essence, a slight random variation or fabrication defect in grating cells would not only be beneficial, but also desirable for many top and bottom grating types up to a variation of 10–15% for most double grating cases. A double grating case, such as top and bottom gratings, usually optimize separately, and a small local perturbation could result in more matching gratings and introduce local light extraction improvement.

Randomization of grating cells increases the light extraction efficiency while having the added benefit of alleviating some of the fabrication complexities demanded by strict periodicities in photonic crystal LEDs. We believe the LED error grating model presents a unique model to analyze fabrication defects associated with laser positioning error and randomizations from chemical etching.

## 3. Results

### 3.1. Top ITO Layer with/without Grating Using Standing Wave Analysis

**Figure 3:**
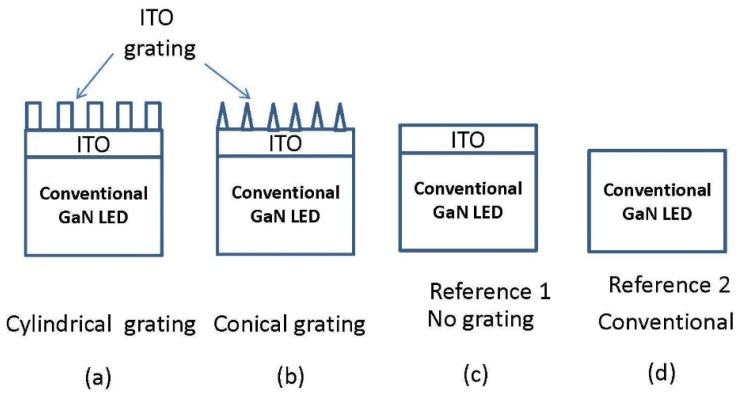
The schematic diagrams of the top grating simulation of (**a**) cylindrical pillar grating, (**b**) conical pillar grating, (**c**) conventional LED (CLED) with Indium Tin Oxide (ITO) layer, and (**d**) conventional LED.

In our ITO nano-grating device design, it is very important to keep a layer of ITO at the bottom of the grating. This fixed thickness is used to prevent the P-GaN layer from being damaged in the etching process and protect the overall device charaterization. Furthermore, the ITO layer also acts as the current injection layer to protect the LED I-V charaterization from being affected by the nano-structure. The ITO top grating studied is shown in Figure 3. In this paper, we focus on the conical ITO study. More simulation results regrading cylindrical top ITO can be found in reference [33].

Standing waves in an LED can either increase or decrease light output from an LED by constructively or destructively interfering at the top surface. By changing the thicknesses of the ITO and sapphire layers, we show that standing wave interference patterns exist in the LED. Studying this effect shows how much the standing wave pattern can either increase or decrease the light extraction from a chip. It is an important step in forming a complete light optimization study of a GaN LED, before any grating will be implemented on top of them, such as top ITO grating, and bottom nano Patterned Sapphire Substrates (PSS) [10].

**Figure 4:**
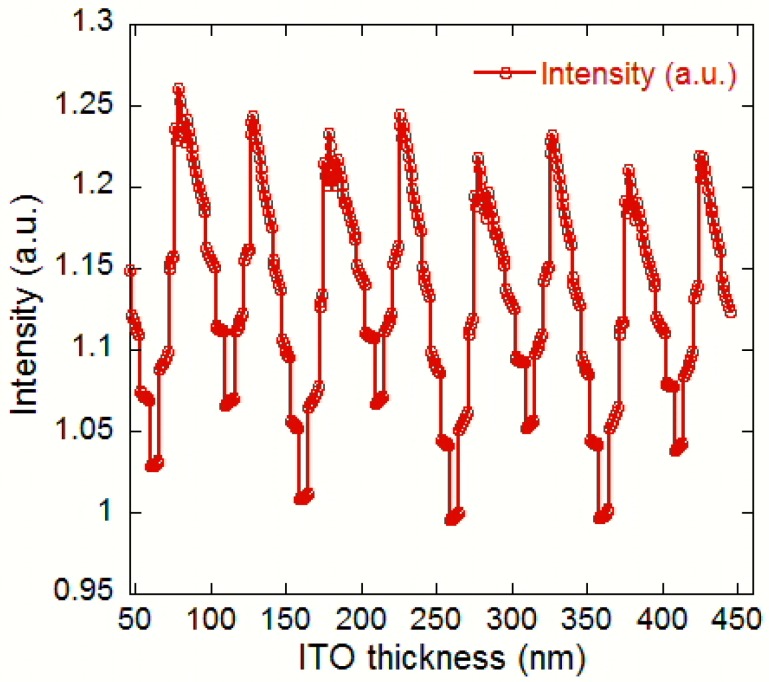
LED top output intensity as ITO thickness varies from 0 nm to 450 nm at 1 nm increments.

We first chose to incrementally add ITO to the surface of the conventional LED (CLED) at a 460 nm wavelength, studying reference 1 in Figure 3c. If no standing wave is present, we should observe a linearly decreasing light output as the ITO is added. However, if a standing wave does exist, adding ITO should cause the output to sinusoidally fluctuate with a linear decrease in output intensity, as shown in Figure 4. As ITO is added, a roughly sinusoidal standing wave pattern emerges whose peak output intensity gradually decreases with increasing material thickness. From Figure 4, the standing wave period is 50 nm and one of the best-case ITO thicknesses occurs at 78 nm, the first constructive interference peak, while one of the worst-case ITO occurs at the 260 nm thickness. This yields a total light increase of 26.7% when the LED is changed from the worst- to best-case ITO thickness. The best-case values are fairly similar as well, from 1.26 a.u. to 1.23 a.u. as the ITO thickness increases. The more accurate values are summarized in Table 2, which presents the light output improvement by 9.6% over the conventional LED (reference 2). The 46 nm ITO thickness is a neutral case, with no improvement and no degradation.

Based on the ITO layer thickness study, the grating period is swept from 92 nm to 920 nm so we can find the grating period that maximizes the top light extraction. We chose this range for the grating because it sweeps the height and width of the cones in the grating from λ/10 to λ. The grating fill factor was held at 0.5 and the ratio of the cone height to the cone width was kept at 1 throughout the study to focus on the effect of the grating period and standing waves on the light output. Because the standing wave analysis showed material thicknesses for the ITO layer that both maximize and minimize light extraction before gratings are added, we studied the grating output at those key ITO thicknesses. As material thicknesses change, the grating output was compared to the instance of reference 1, that has the same ITO thickness. However, reference 2, the conventional LED, was used as an unchanging reference and its sapphire substrate thickness remains fixed at 10,000 nm in here to reduce the simulation time and space.

In our earlier study [31], we used a two-dimensional (2D) rigorous couple wave analysis (RCWA) GaN LED grating model to study top diffraction gratings, and compare none-grating, cylindrical-grating, and conical-grating cases. It showed that the cylindrical grating has better performance. Therefore, our study is focused on the conical nano-ITO grating case, and compares it with the none-grating (reference 1) and conventional LED (reference 2) [10].

**Figure 5:**
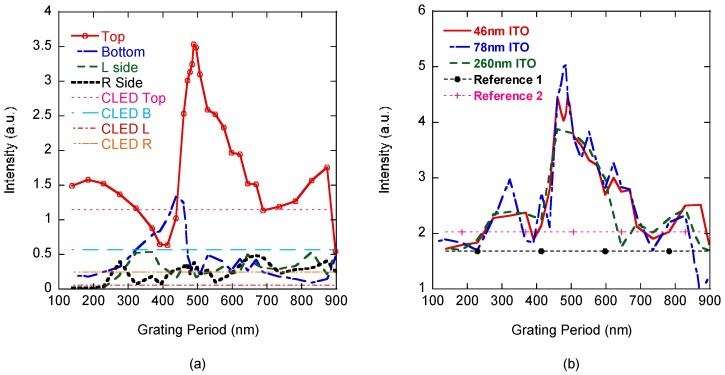
Light extracted from LED as the grating period is varied. (**a**) Light extraction from four monitors (top, bottom, left side, and right side) compared with conventional LED (reference 2) for an ITO thickness of 46 nm; (**b**) total light extraction for difference ITO thicknesses and compared with reference 1 with an ITO of 46 nm and reference 2.

Understanding where light is emitted is important, because some systems are designed to capture and direct light emitted from the sides and bottom of the LED. For these systems, maximizing total light output may be more important. Figure 5a shows that most of the light is emitting through the top, and is also most sensitive to the grating design, and the bottom is second. Side light emittance is very low and very insensitive to the grating implementation. Compared to the conventional LED (reference 2), top emittance also has the highest improvement, about 204% for both reference 1 and 2. Bottom light emittance for the best grating case can reach a 241% and 132% improvement according to reference 1 and reference 2, respectively. Left and right monitor simulations have some difference, which comes from slightly different monitor location placements. We also calculate reference 1 and reference 2’s monitor outputs, as listed in Table 3 and calculated the total light extraction (top + bottom + left side + right side) according to the grating period variation (Figure 5b). It showed that gratings can significantly improve light output around the 500 nm grating period and increase light output at most periods. However, the results also show that the grating can reduce light output by more than 50% if a period of 400 nm or 900 nm is used. As expected, the grating on the best case ITO layer (78 nm) has the highest light output at 5.03 a.u., resulting in an approximately 199% improvement for reference 1 and 148% improvement for reference 2.

### 3.2. Nano-patterned Sapphire Substrates (PSS) Bottom Grating, SiO_2_ Nano-rod Grating (NR), and Ag Reflector

We studied the conical bottom reflection grating and published top/bottom grating design simulation in reference [5]. The simulation results show that simple or direct combinations of the optimized top grating with the optimized bottom grating only produces a 42% light extraction improvement compared to the non-grating conventional LED, which is much lower than that of an optimized single grating case (about 165%). This is due to the mismatch of two grating parameters with the direct addition of the second grating structure, which changes optical modes in the LEDs. Therefore, it is very important to optimize both top and bottom gratings simultaneously for the double-grating design [5]. In this section, we optimized two or three structures simultaneously to achieve the final design comparison.

**Figure 6:**
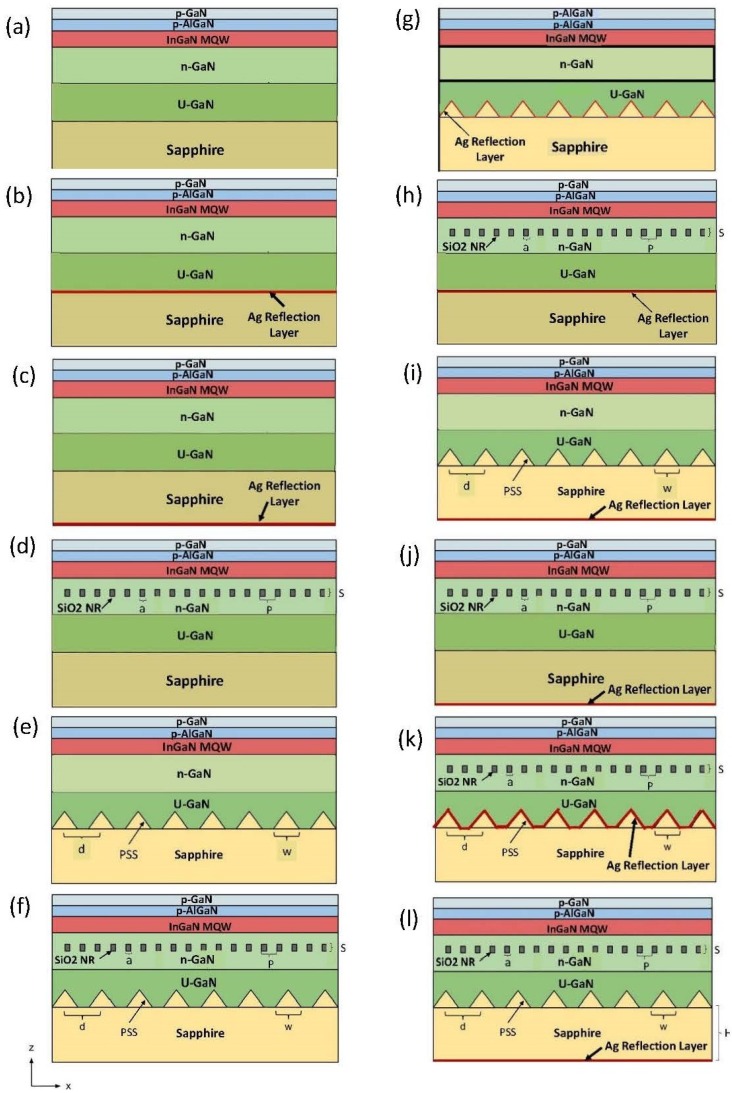
Diagram of LED with a patterned sapphire substrate (PSS), SiO_2_ NR array, and *Ag Reflector*. The diagram defines the PSS period (*d*), PSS width (*w*), SiO_2_ NR period (*p*), SiO_2_ NR width (*a*), SiO_2_ NR high (*s*), and shows the *x* and *z* direction of the LED. (**a**)–(**l**) are explained and listed in the paper.

Most nano-bottom gratings are fabricated on the sapphire substrate, currently called a patterned sapphire substrate, as shown in Figure 6e. In this section, we simulated GaN LED structures with a patterned sapphire substrate and an embedded SiO_2_ nanorod array. To confirm the accuracy of our software, we simulated a conventional LED (CLED) and PSS NR LED. Neither of these two structures contained an Ag reflection layer. Previous experiments have revealed that adding a patterned sapphire substrate and SiO_2_ nanorod array increases the external quantum efficiency by 48% [34] or 56% [25] compared to CLED. Our corresponding simulation results show that the PSS NR structure increases light extraction by 52% compared to the CLED, from CLED 32.239 a.u. to PSS NR 48.925 a.u. These results agree very well with the published experimental data. Next, we optimized PSS by changing the period (*d*) from 2 μm to 3 μm in steps of 0.5 μm and width (*w*) from 1 μm to 2.5 μm in steps of 0.1 μm. A more stable point was obtained where *w* = 2.5 μm and *d* = 3 μm, which will be used in our later simulation. The more detailed results for various grating widths and periods for PSS are published in reference [35] and is not the focus of this paper. We also optimized the position of the SiO_2_ NR array by moving it up and down in the z direction between 7 μm and 8 μm, and chose a maximum light output location of *z* = 7.8 μm for the final simulation.

Based on the basic simulation results, we fully investigated 12 state of the art LED structures, which include (Figure 6):

(a) Conventional LED (CLED);
(b) conventional LED with Ag between U-GaN and a sapphire substrate;
(c) conventional LED with Ag below a sapphire substrate;
(d) LED with an SiO_2_ nanorod (NR)) array;
(e) LED with a patterned sapphire substrate (PSS);
(f) LED with a PSS and NR array (PSS NR);
(g) LED with PSS, Ag between U-GaN, and a sapphire substrate;
(h) LED with an NR array, Ag between U-GaN, and a sapphire substrate;
(i) LED with PSS, Ag below a sapphire substrate;
(j) LED with NR array, Ag below a sapphire substrate;
(k) LED with PSS, NR array, and Ag between U-GaN and a sapphire substrate; and
(l) LED with PSS, NR array, and Ag below a sapphire substrate.

Combinations of PSS and NR arrays have shown to increase crystal quality by reducing thread dislocations, and increase external quantum efficiency by scattering light [34]. We chose to simulate this structure because of its high efficiency. The conventional LEDs were simulated as a reference point. All simulations were compared with the conventional LED. If a structure cannot perform better than a conventional LED and is more difficult to fabricate, it may not be worth fabricating. Figure 6 does not show two key components of our simulation, the excitation source located at the MQW region and the time monitor located just above the p-GaN layer. The excitation source emits light at a wavelength of 460 nm, while the monitor records the average power extracted. Table 4 below provides a description of the conventional LED, including the substrate material, thickness, and refractive index, for this comparison simulation.

**Figure 7:**
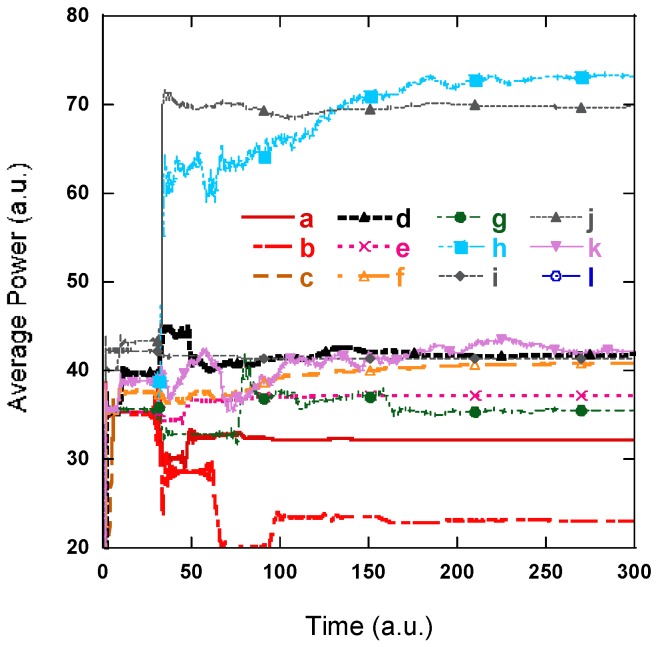
Average power of light extracted in 10 structures, while the PSS width, *w* = 2.5 μm, and period, *d* = 3 μm. The SiO_2_ layer is centered at *z* = 7.8 μm. Sapphire layer thickness is 80 μm. (**a**) CLED, (**b**) CLED with Ag between U-GaN and the sapphire substrate, (**c**) CLED with Ag below the sapphire substrate, (**d**) NR, (**e**) PSS, (**f**) PSS NR, (**g**) PSS with Ag between U-GaN and the sapphire substrate, (**h**) NR with Ag between U-GaN and the sapphire substrate, (**i**) PSS, Ag below the sapphire substrate, (**j**) NR array, Ag below the sapphire substrate, (**k**) PSS NR array with Ag between U-GaN and the sapphire substrate, and (**l**) LED with PSS, NR array, and Ag below the sapphire substrate.

**Figure 8:**
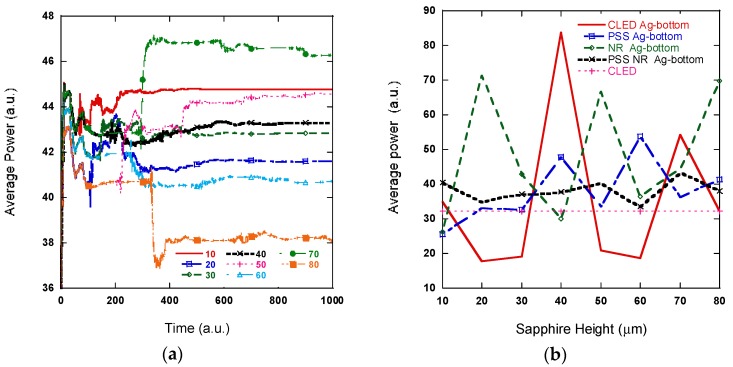
Average power vs. different sapphire substrate thicknesses with PSS NR and a bottom layer Ag reflector. PSS width of *w* = 2.5 μm and period of *d* = 3 μm. SiO_2_ layer is centered at *z* = 7.8 μm. (**a**) Average power extracted against time according to different sapphire substrate thicknesses of 10 μm, 20 μm, 30μm, 40 μm, 50 μm, 60 μm, 70 μm, and 80 μm, and (**b**) steady state average power.

The results in Figure 7 lead to several observations. First, Figure 7 show that both PSS and SiO_2_ nano-rod structures enhance light extraction; a 15% improvement for the PSS structure and a 26% improvement for the PSS and SiO_2_ NR array structure. The structure with both the PSS and SiO_2_ nano-rod array increases light extraction more than the simulation only containing the PSS. This is because that SiO_2_ NR structure alone can improve light extraction by 30%, which is higher than PSS only or PSS NR. Second, adding Ag reflection may not necessarily improve light extraction, as shown in the CLED Ag-middle and Ag-bottom cases. Inappropriate placement of the Ag layer may cause a destructive interference pattern or force minimum standing wave output at the top of the LED. Therefore, this may decrease the output light intensity. The third, NR with an Ag reflector improves light extraction by about 127% for Ag-middle and 116.38% with Ag-bottom, which are the best cases for the 12 designs. It is very worthwhile to fabricate NR with an Ag reflector structure. Fourth, the output power of the structures containing the bottom Ag reflection layer fluctuates with the sapphire substrate thickness because of the standing wave pattern inside the LED cavity [35], as shown in Figure 8. Our simulation also shows that the sapphire substrate height (H) does not affect structures without Ag reflectors or Ag reflectors above it. The conventional LED with the reflection layer shows the greatest change in output power. At a height of 40 μm, light extraction increases by 160% and at a height of 20 μm, light extraction decreases by 45% compared to the CLED structure, which can also be understood by the standing wave analysis.

A summary of the results, including the percent improvement compared to the conventional LED, are listed below in Table 5. For the three kinds of structures investigated, NR is the most efficient and intensively studied. PSS is a reasonable method to improve the light extraction efficiency. An Ag-reflector can improve or decrease light output, because it can change the standing wave pattern inside an LED dramatically and cause huge effects. When designing carefully, an NR plus Ag-reflector can be one of the best designs of GaN LEDs.

### 3.3. Nano-top Grating Performance over Different Wavelength

**Figure 9:**
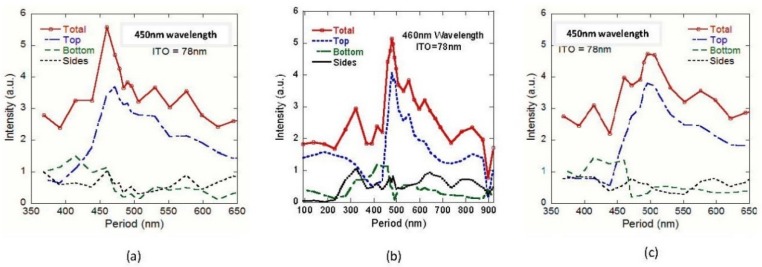
Total light output for ITO nano-top grating when the free space wavelength is at (**a**) 450 nm, (**b**) 460 nm, and (**c**) 470 nm.

In reality, regarding LEDs’ output light over a range of wavelengths surrounding the primary wavelength, we verified that the grating period that maximizes light extraction at the center wavelength is still effective at other wavelengths in the primary emission range. To better understand how the wavelength of light affects light output, the grating periods around the peak light emission were re-simulated for the ITO nano-top grating at free space wavelengths of 470 nm and 450 nm, as shown in Figure 9. These are wavelengths that are near the peak wavelength of 460 nm and are still strongly emitted by the LED. These simulations use an ITO thickness of 78 nm and a sapphire substrate thickness of 10,050 nm.

It shows that the gratings are still highly effective at increasing the light output from the CLED for both 450 nm light and 470 nm light. Additionally, total light extraction, with a wavelength of λ_o_ = 450 nm, is substantially higher than any total light extraction that was achieved for either the 460 nm or 470 nm light. This is because the shoulder of the top emission peak overlaps with a sharp rise in light extraction from both the bottom and sides of the LED. The top light extraction efficiency is still highest for the 460 nm wavelength, which is our design wavelength. The most effective grating period in all three wavelengths is about 500 nm, which is our next simulation parameters.

**Figure 10:**
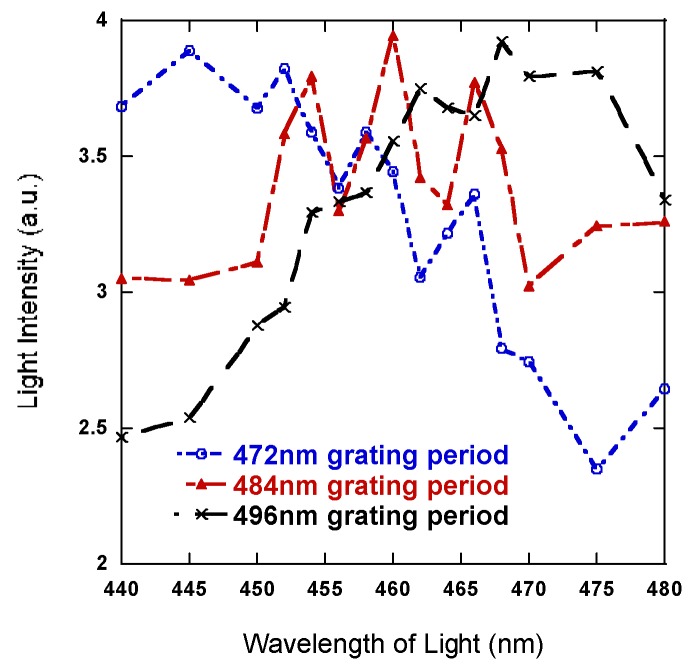
Light extraction intensity across the LED emitting spectrum.

The maximum output for each wavelength occurs at a different grating period, but all are a little smaller and close to 500 nm. It shows that for maximum top light extraction, shorter wavelengths correlate with smaller grating periods and longer wavelengths correlate with bigger grating periods [10]. Because the material layers were optimized for 460 nm light in the standing wave section, the larger grating periods, like 496 nm, enhance the longer wavelengths of light within the LED emitting spectrum and shorter periods around 472 nm enhance the shorter wavelengths, which is clearly shown in Figure 10 as well. The grating period that maximizes total light output for our design is a grating period near 484 nm.

## 4. Conclusions

We proposed an error grating model to simulate the fabrication variation and its effects. All simulations were based on the FDTD method. The scope of this research covers the effects of nano-scale top gratings, patterned sapphire substrates, SiO_2_ nano-rod arrays, and Ag reflection layers.

We investigated the effect of nanoscale ITO transmission gratings on light emission from the top, the side, and the bottom of a GaN LED based on substrate standing wave analysis. Standing wave analysis of the LED showed that the light extraction efficiency can be improved by varying the ITO thickness, and optimized the ITO grating parameters, which can reach a 204% improvement compared to CLED. We also optimized light extraction for a structure containing a patterned sapphire substrate and an SiO_2_ nano-rod array. Both PSS and SiO_2_ nano-rod structures enhanced light extraction. A 15% improvement for the PSS structure and a 30% improvement for NR was achieved. A combination of NR and PSS may not be a better design, with a 26.459% improvement here. A more careful design simulation is needed to obtain the overall optimizing results. We also found that the position of the reflection layer affected the light extraction. From these simulations, we found a maximum increase in light extraction of 127% for an SiO_2_ nano-rod LED with an Ag reflection layer compared to a conventional LED. The LED with the NR and Ag reflector structure was the best design.

Finally, we studied the nano-top grating performance over different wavelengths to validate our design simulation and generate an LED emitting spectrum. The grating period had clear influence on the emitting spectrum. However, our design was still reasonable between 440 nm and 480 nm around the center wavelength of 460 nm.

## Figures and Tables

**Table 1:** LED material thickness and refractive indices at λ = 460 nm.

Material	Sample Thickness (μm)	Refractive Index
ITO	0.23	2.1
p-GaN	0.2	2.5
InGaN/GaN MQWs	0.1	2.6
n-GaN	2	2.5
GaN	3	2.5
Sapphire	80	1.78
Ag	Reflection: 90%	

**Table 2:** Light output intensities at the best, worst, and neutral ITO thicknesses.

ITO Thickness	Output Intensity (a.u.)	% Improvement over CLED
46nm	1.1500	0.000
78nm	1.2606	9.617
260nm	0.9946	−13.513

**Table 3:** Light output intensities at difference monitors for ITO 46nm case and refrernces.

Monitor	Intensity (a.u.) reference 1	Intensity (a.u.) reference 2	Intensity (a.u.) for ITO 46 nm maximum value	Intensity (a.u.) for ITO 46 nm grating period 500 nm
Left	0.017	0.0605	0.533	0.16
Right	0.124	0.244	0.488	0.3
Bottom	0.3897	0.5726	1.322	0.20
Top	1.149	1.15	3.5778	3.54
**Total**	**1.680**	**2.027**	**------**	**4.20**

**Table 4:** Conventional GaN LED Parameters.

Material	Thickness (μm)	Refractive Index
p-GaN	0.12	2.55
P-AlGaN	0.05	2.5
InGaN/GaN	0.115	2.6
n-GaN	2	2.55
GaN	3	2.55
Sapphire	80	1.77

**Table 5:** Percent improvement of various LEDs compared to CLEDs.

	Structure	Average Power (a.u.)	Percent Improvement (%)
**a**	Conventional (CLED)	32.239	---
**b**	CLED Ag-middle	23.078	−28.416
**c**	CLED Ag-bottom	32.227	−0.037218
**d**	NR (only)	41.934	30.072
**e**	PSS (only)	37.159	15.261
**f**	PSS & NR	40.769	26.459
**g**	PSS Ag-middle	35.461	9.9941
**h**	NR Ag-middle	73.191	127.03
**i**	PSS Ag-bottom	41.358	28.29
**j**	NR Ag-bottom	69.759	116.38
**k**	PSS NR Ag-middle	42.221	30.963
**l**	PSS NR Ag-bottom	38.011	17.904
